# Shot-to-shot two-dimensional photon intensity diagnostics within megahertz pulse-trains at the European XFEL

**DOI:** 10.1107/S1600577522005720

**Published:** 2022-06-08

**Authors:** Trey W. Guest, Richard Bean, Johan Bielecki, Sarlota Birnsteinova, Gianluca Geloni, Marc Guetg, Raimund Kammering, Henry J. Kirkwood, Andreas Koch, David M. Paganin, Grant van Riessen, Patrik Vagovič, Raphael de Wijn, Adrian P. Mancuso, Brian Abbey

**Affiliations:** a La Trobe Institute for Molecular Science, La Trobe University, Bundoora, VIC 3086, Australia; b Department of Mathematical and Physical Sciences, School of Engineering, Computing and Mathematical Sciences, Bundoora, VIC 3086, Australia; c European XFEL, Holzkoppel 4, 22869 Schenefeld, Germany; d Deutsches Elektronen-Synchrotron, Notkestrasse 85, 22607 Hamburg, Germany; e School of Physics and Astronomy, Monash University, VIC 3800, Australia

**Keywords:** X-ray free-electron lasers, photon diagnostics, source characterization, European XFEL, MHz XFEL, XFEL radiation, beam imaging

## Abstract

Characterizing the properties of X-ray free-electron laser (XFEL) sources is a critical step for optimization of performance and experiment planning. The recent availability of MHz XFELs has opened up a range of new opportunities for novel experiments but also highlighted the need for systematic measurements of the source properties. Here, MHz-enabled beam imaging diagnostics developed for the SPB/SFX instrument at the European XFEL are exploited to measure the shot-to-shot intensity statistics of X-ray pulses. The ability to record pulse-integrated two-dimensional transverse intensity measurements at multiple planes along an XFEL beamline at MHz rates yields an improved understanding of the shot-to-shot photon beam intensity variations. These variations can play a critical role, for example, in determining the outcome of single-particle imaging experiments and other experiments that are sensitive to the transverse profile of the incident beam. It is observed that shot-to-shot variations in the statistical properties of a recorded ensemble of radiant intensity distributions are sensitive to changes in electron beam current density. These changes typically occur during pulse-distribution to the instrument and are currently not accounted for by the existing suite of imaging diagnostics. Modulations of the electron beam orbit in the accelerator are observed to induce a time-dependence in the statistics of individual pulses – this is demonstrated by applying radio-frequency trajectory tilts to electron bunch-trains delivered to the instrument. We discuss how these modifications of the beam trajectory might be used to modify the statistical properties of the source and potential future applications.

## Abstract 2

Systematic fluctuations in the pulse–pulse intensity profile of a MHz X-ray free-electron laser (FEL) source have been observed that are not described by fundamental FEL theory. The sensitivity of these fluctuations to modifications of the electron-beam orbit in the accelerator is demonstrated as a potential route to beam optimization.

## 1. Introduction

X-ray free-electron laser (XFEL) light sources have enabled fundamental advancements in X-ray science due to their extreme brightness and ultrashort pulse duration (Rossbach *et al.*, 2019 ▸). Self-amplified spontaneous-emission (SASE) XFEL sources have been demonstrated to achieve up to ten orders of magnitude increase in peak spectral brightness in comparison with third-generation synchrotrons (Saldin *et al.*, 2000 ▸). This increase in source brilliance addresses some of the inherent limitations of synchrotron radiation sources and electron microscopes (Spence *et al.*, 2012 ▸). The use of extremely intense, femtosecond-duration X-ray pulses opens up the possibility of ‘out running’ radiation-induced damage which can degrade the quality and/or resolution of diffraction measurements (Neutze *et al.*, 2000 ▸; Abbey *et al.*, 2016 ▸; Ryan *et al.*, 2017 ▸). Some of the most exciting potential applications of XFELs include single-particle imaging (SPI) (Sobolev *et al.*, 2020 ▸) and MHz-rate high-contrast full-field microscopy (Vagovič *et al.*, 2019 ▸). These techniques have enabled the observation of dynamically evolving biological or materials systems which cannot typically be captured using conventional crystallography or electron microscopy (Bielecki *et al.*, 2020 ▸; Darmanin *et al.*, 2016 ▸). The source characteristics of XFELs are still an active area of research, particularly in the case of recently developed high-repetition-rate facilities such as the European XFEL, that can deliver tens of thousands of SASE pulses per second. The source properties of these MHz XFELs are potentially quite distinct from those of their lower-repetition-rate counterparts due to electron-beam conditioning and distribution effects that are not related to the fundamental shot-noise fluctuations (Yefanov *et al.*, 2019 ▸).

Characterizing the properties of XFEL sources is critical for optimization of performance and experiment planning. The recent availability of MHz XFELs has opened up a range of opportunities for novel experiments but also highlighted the need for systematic measurements of the source properties to be performed. Here we exploit MHz-enabled two-dimensional imaging diagnostics developed at the European XFEL to measure the shot-to-shot intensity statistics of SASE pulses. While single-shot photon diagnostics have been developed to monitor the position (Tono *et al.*, 2011 ▸) and intensity (Roth *et al.*, 2018 ▸) of XFEL pulses, no method of measuring the two-dimensional shot-to-shot intensity profile of a MHz XFEL source has been demonstrated. Recording the pulse-integrated two-dimensional transverse intensity of MHz XFEL pulses will enable an improved understanding of the source characteristics and statistical properties of the photon beam. An analysis of these variations will provide new insights into the fundamental properties of MHz XFEL sources and can potentially help with experimental planning leading to more successful outcomes.

The Single Particles, Biomolecules and Clusters/Serial Femtosecond Crystallography (SPB/SFX) instrument sits in the direct branch of the SASE1 undulator beamline of the European XFEL (Abeghyan *et al.*, 2019 ▸). The electron-bunch current from SASE1 can be delivered to SPB/SFX and its neighbouring instrument in flexible bunch patterns using dedicated electron bunch-distribution systems (Obier *et al.*, 2019 ▸). The SPB/SFX instrument was primarily designed for the 3D structural determination of crystalline and non-crystalline micrometre-scale and smaller biological objects (Mancuso *et al.*, 2019 ▸). A pair of horizontal offset mirrors for photon transport are located in the photon tunnel 260 m downstream of the undulator (Sinn *et al.*, 2011 ▸). SPB/SFX is located approximately 900 m downstream of SASE1 – the nanofocus configuration of the instrument optics contains a pair of Kirkpatrick–Baez (KB) mirrors (Bean *et al.*, 2016 ▸) for independent focusing along each of the transverse axes. The instrument is optimized for peak performance at 6–15 keV and supports sample-to-detector distances between 120 mm and 6000 mm for inline scattering experiments. More recently, a direct beam configuration allows the beamline to reach energies as high as 24 keV for MHz microscopy applications.

The European XFEL is the world’s first hard X-ray MHz repetition-rate XFEL light source and part of its mission is to address the fundamental limitations due to the high data-volume required for SFX and SPI experiments (Grünbein *et al.*, 2018 ▸; Wiedorn *et al.*, 2018 ▸). To date, there has only been a limited amount of work carried out investigating photon beam properties at the European XFEL that occur at MHz shot-to-shot repetition rates. In part, this is due to the lack of experimental tools capable of recording the properties of high-photon-energy XFEL pulses at MHz frame-rates. Current beam-profile diagnostics used for beam alignment at the European XFEL operate at frame-rates equivalent to the inter-train frequency of 10 Hz (Koch *et al.*, 2019 ▸) and are therefore not applicable to the study of fluctuations at MHz rates. Here we exploit the recently developed MHz X-ray imaging diagnostics tools now available at the SPB/SFX instrument in order to investigate the shot-to-shot two-dimensional intensity statistics of pulses produced from the SASE1 undulator (Grünert *et al.*, 2019 ▸).

## 2. Pulse-delivery at the European XFEL

**Figure 1:**
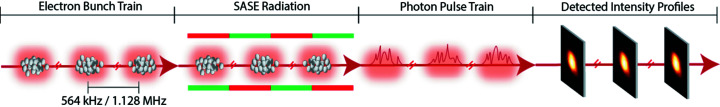
European XFEL pulse structure incorporating the MHz imaging diagnostics measurement system. The schematic illustrates the relationship between the electron bunches, delivered in bunch-trains, and subsequent photon pulses and photon pulse-trains, which are recorded as two-dimensional intensity profiles after propagation.

The burst-mode of operation at the European XFEL (Fig. 1 ▸) can be considered with reference to three timescales: (i) the timescale of the duration of individual XFEL pulses, (ii) the inter-pulse timescale within a given train (*i.e.* intra-train) and (iii) the inter-train timescale. The properties of individual SASE pulses (10–100 fs duration) cannot be characterized directly using current 2D detectors; only the integrated intensity profile of each pulse can be measured via an X-ray sensitive scintillator and fast optical camera (Vagovič *et al.*, 2019 ▸). X-ray pulses can be delivered to the instrument at a maximum inter-pulse repetition rate of 4.5 MHz (Altarelli, 2006 ▸). Electron bunch-train bursts containing up to 2700 bunches can be injected into the accelerator, although the number of pulses delivered to the instrument in an experimental context is typically detector limited (Allahgholi *et al.*, 2019 ▸). Each pulse-train is delivered to the instrument at a repetition rate of 10 Hz.

We measure the two-dimensional intensity profile of individual XFEL pulses by recording the integrated optical decay profile of a femtosecond excited 100 µm-thick YAG:Ce scintillator using a fast-frame-rate Shimadzu HPV-X2 high-speed optical camera coupled with a motorized zoom objective. The integrated intensity of the photon beam was recorded over the duration of each individual XFEL pulse. The exposure function is dominated by the nanosecond time constant of the YAG:Ce scintillator (Moszyński *et al.*, 1994 ▸). The exposure time of the high-speed optical camera was nominally 550 ns and was synchronized to the arrival time of the beam. The beam profile is recorded as a set of two-dimensional intensity distributions at the variable frame-rate of the optical camera. In the present case the inter-pulse and inter-train beam profiles are defined as the intensity distributions integrated over individual pulses and pulse-trains, respectively. We assign each of the pulses an index within their respective pulse-trains (intra-train) which corresponds to the order in which the electron bunches arrive within their respective electron-bunch trains. This pulse-index is reset at the beginning of each pulse-train. Inter-train beam profiles are comparable with those obtained using non-MHz-enabled imaging diagnostics.

The rate of change in intensity along the optical axis is proportional to the local tilt and curvature of the wavefield, which manifest as the displacement and demagnification of the recorded optical intensity (Teague, 1983 ▸). We record the change in beam radius, Δ*r*, and transverse centre of mass, Δ*x*/Δ*y*, of the detected two-dimensional beam intensities relative to the mean value of the average intensity profile of the recorded ensemble. The beam radius was calculated by fitting a circle with an origin at the centre-of-mass of the intensity distribution that encloses a fraction of the total pulse intensity. The enclosed region was equivalent to the energy fraction contained within the full width at half-maximum (FWHM) of a Gaussian distribution. Pulses were recorded at an inter-train frame rate of 10 Hz, as well as intra-train frame rates of 564 kHz and 1.128 MHz, which correspond to the sub-harmonic frequencies of the maximum intra-pulse repetition rate of the instrument. In the 10 Hz case, fluctuations in the recorded intensity profiles are compared with the mean beam-size and position of the train-integrated pulse-ensemble, while at 564 kHz and 1.128 MHz these figures of merit are presented relative to the global mean (*i.e.* the mean of all pulses) and the intra-train mean. Photon pulse energy was recorded using X-ray gas monitors (XGMs) located upstream and downstream of the tunnel optics.

## 3. Inter-pulse properties and pulse distribution

**Figure 2:**
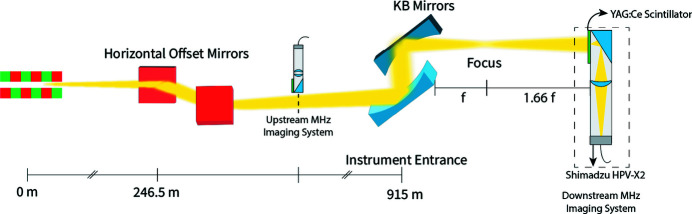
Schematic of the MHz beam imaging diagnostics experiment using the nanofocus setup at the SPB/SFX instrument of the European XFEL.

Firstly, we explore changes in the shot-to-shot properties of the beam at MHz frame rates for two bunch distribution patterns containing 50 and 100 SASE pulses, delivered at 564 kHz and 1.128 MHz, respectively. White-field exposures (*i.e.* in the absence of a sample) of 6 keV XFEL pulse-trains were taken at an inter-train frame-rate of 10 Hz and intra-train frame-rates of 564 kHz and 1.128 MHz. A Navitar Resolv4K objective was used at 1.1 magnification to achieve an effective pixel-size of 29 µm. The centre-of-mass and radius of each two-dimensional intensity distribution was determined for X-ray pulse-trains downstream of the focus. Pulse-trains were sampled approximately every 150 trains (15 s), which was determined by the duty cycle of the detector. An illustration of the optical setup is shown in Fig. 2 ▸.

**Figure 3:**
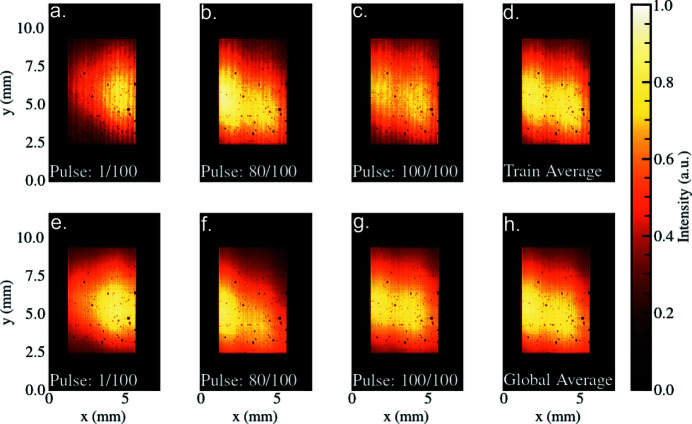
MHz resolved transverse intensity distributions recorded downstream of the SPB/SFX focal plane for a subset of inter-train positions 1, 80 and 100 (*a*–*c*) over a single pulse train, and (*e*–*g*) integrated over all recorded pulse-trains. The train- and global-averages of the recorded pulse-profiles are given in (*d*) and (*h*), respectively.

A summary of the inter- and intra-train statistics of the recorded pulses is given in Table 1 ▸. The inter-pulse intensity profile of the XFEL beam recorded by the MHz-enabled imaging diagnostics is presented in Fig. 3 ▸ for a subset of pulse-positions.

**Figure 4:**
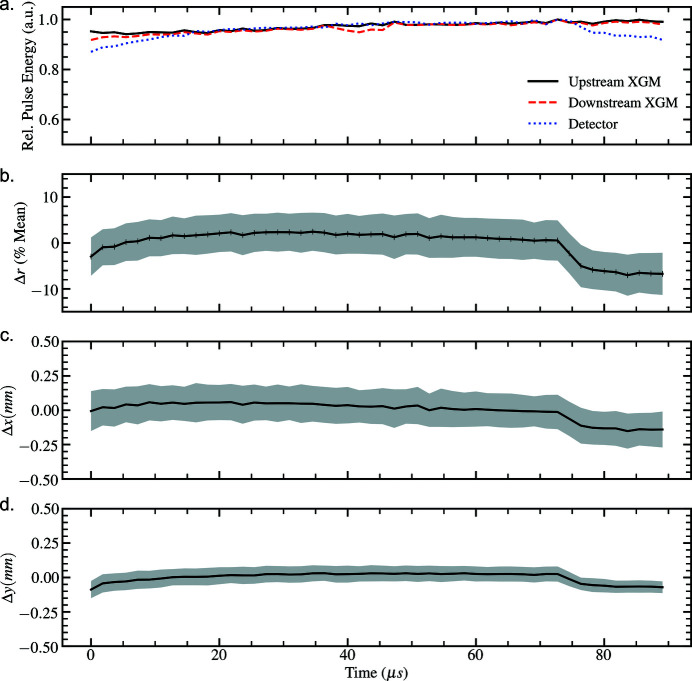
MHz intensity diagnostics illustrating fluctuations in (*a*) pulse-energy, (*b*) magnification/demagnification, (*c*–*d*) horizontal and vertical beam displacement, averaged over all pulse-trains as a function of pulse position for the 50 pulse-per-train bunch-structure delivered at 564 kHz. Shaded regions denote ±1 standard deviation from the average measurement.

The shot-to-shot statistical properties of the recorded intensity distributions are presented as a function of time (for a given train) for the 50 pulse-per-train and 100 pulse-per-train bunch structures in Figs. 4 ▸ and 5 ▸. The intra-train beam properties show a dependence of both beam position and size on the position of the pulse within a pulse-train in both cases. In all cases, the transverse position of the beam exhibits a systematic drift until an event, likely due to electron beam-distribution between branches of the SASE1 beamline, at approximately 67 µs, corresponding to the 40th and 80th pulses in the respective pulse-trains, which leads to rapid variation in the properties of the photon beam. Henceforth we refer to the systematic drift and rapid pulse-to-pulse variations as corresponding to ‘slow’ and ‘fast’ timescales, respectively, in the recorded intensity distributions.

**Figure 5:**
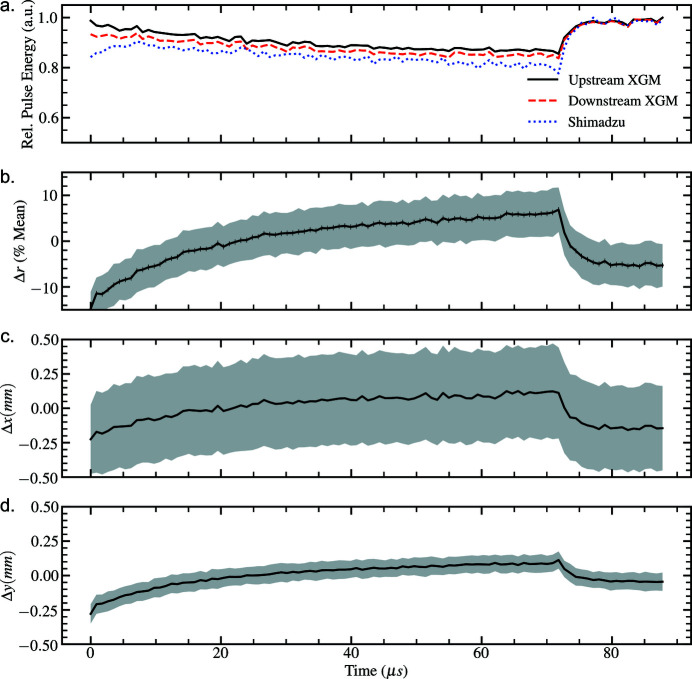
MHz intensity diagnostics illustrating fluctuations in (*a*) pulse-energy, (*b*) magnification/demagnification, (*c*–*d*) horizontal and vertical beam displacement, averaged over all pulse-trains as a function of pulse position for the 100 pulse-per-train bunch-structure delivered at 1.128 MHz. Shaded regions denote ±1 standard deviation from the average measurement.

Movie 1 in the supporting information depicts the intra-train evolution of the average two-dimensional intensity profile of the beam at each pulse position at 1.128 MHz. We observe that the slow-timescale displacement of the beam can be identified as both (i) the relocation of intensity between lobes of the beam profile, which is due to photon transport optics in the tunnel, as well as (ii) the transverse displacement of the beam across the upstream optics. The shape of the intra-train intensity distribution is more stable for the 564 kHz scenario (Movie 2 in the supplementary information), which may be due to the reduced size of the 50 pulse-per-train intensity distribution at the tunnel optics (Samoylova *et al.*, 2009 ▸). In each case, the fast-timescale beam correction manifests as a rapid relocation of the beam intensity. Measurement of this fast timescale event via the upstream XGM in the photon-tunnel, prior to photon transport optics (Fig. 5 ▸), suggests that the recorded perturbation in beam intensity may arise from changes in the machine that occur intra-train.

## 4. Inter-pulse properties and electron beam trajectory

**Figure 6:**
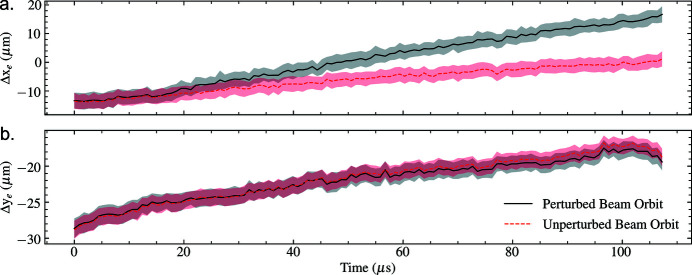
Transverse train-averaged shot-to-shot electron bunch displacement for the perturbed and unperturbed electron-beam trajectories in each of the transverse directions: (*a*) horizontal, (*b*) vertical. Shaded regions denote ±1 standard deviation from the average measurement.

**Figure 7:**
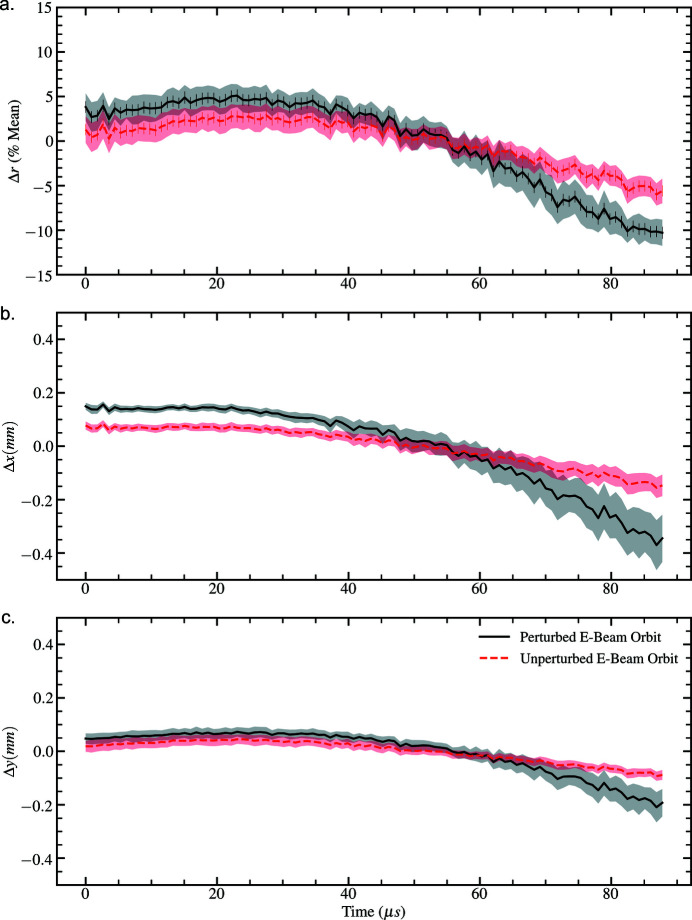
MHz intensity diagnostics depicting fluctuations in (*a*) magnification/demagnification and (*b*–*c*) horizontal and vertical beam displacement averaged over all pulse-trains as a function of pulse position for photon beams corresponding to the perturbed and unperturbed electron bunch train trajectories. Shaded regions denote ±1 standard deviation from the average measurement.

Dedicated short-pulse (∼50 ns) and long-pulse (∼300 ms) electromagnetic kicker arrays control the electron-bunch pattern delivered for lasing into the three operational undulators at the European XFEL. While the kicker arrays are typically used to select individual electron-bunches and bunch-trains for lasing, here we use a single long-pulse kicker to apply a 15 µm linear slope to the horizontal trajectory of each bunch-train and assess the sensitivity of the inter-pulse properties to this perturbation in the photon tunnel. 128 pulses-per-train were recorded at 1.128 MHz across 122 trains for a 9 keV XFEL beam. An OptoSigma ULWD lens was used at 200× magnification to achieve an effective pixel-size of 13.2 µm. The electron-beam trajectory was recorded using an electron beam position monitor (BPM), and photon intensity diagnostics were collected using a drop-in screen located downstream of the photon transport optics (approximately 300 m upstream of the instrument entrance). We label the beam trajectories as being either perturbed or unperturbed. The transverse beam displacements throughout the electron bunch trains measured by the BPM are presented with respect to electron-bunch number in Fig. 6 ▸. The subsequent intra-pulse properties of the beam were assessed using the previously discussed photon-beam stability metrics and are presented in Fig. 7 ▸.

The inter-pulse photon beam diagnostics indicate a degradation in the stability in terms of the size and position of the two-dimensional spatial intensity distribution of the modified electron beam on the timescale of a single pulse. This is evident in the increased variance in displacement and beam-size of the recorded intensity distributions in comparison with the unmodified beam trajectory, provided in Table 2 ▸. Consequently, we observe that the photon beam pointing change is sensitive to modifications in the electron-beam trajectory in the accelerator; however, no obvious correlation between the electron-bunch train and pulse-train trajectories is seen from a comparison of Figs. 6 ▸ and 7 ▸.

## 5. Discussion

The MHz frame-rate two-dimensional intensity monitors installed at the European XFEL offer the capacity to sensitively measure variations in pulse beam size and position inter- and intra-train. The range of fluctuations in beam size and position is observed to be greater when measured inter-pulse than when recording the train-integrated intensity distribution. This disparity between imaging diagnostics measurements recorded at inter- and intra-train frame-rates indicates that accurate evaluations of pulse stability in XFEL experiments where datasets are constructed over multiple, randomly selected exposures (Ayyer *et al.*, 2021 ▸) may require pulse properties to be assessed using MHz-resolved detectors. It is clear that estimates of shot-to-shot beam size and position fluctuations obtained from train-integrated diagnostics do not accurately reflect the degree of variation as measured on intra-train timescales. Due to the comparatively low sampling rate (one train in 150) of the shot-to-shot diagnostics, we do not consider trends that may potentially occur on the inter-train timescale. The current underestimation of the degree of variance in the inter-pulse photon statistics could potentially have implications for our understanding of the source properties.

We have observed the properties of individual XFEL pulse trains to vary with the number of pulses distributed to the instrument and to a degree that is not adequately represented by train-integrated imaging diagnostics. The average range of variation in pulse characteristics intra-train was observed to be smaller than the range of the variations globally. This is consistent with observations which illustrate fluctuations in the average size and centre-of-mass of the train-integrated beam profile in Movie 3 of the supporting information. These fluctuations may arise from inter-train instability of the electron bunch-trajectory or photon transport optics. In order to develop a functional understanding of the contributions to shot-to-shot variability at the European XFEL, the described MHz photon intensity diagnostics should be applied to systematically assess the contribution of the machine and instrument parameter space to fluctuations in beam size and position intra-pulse as an approximate measure of beam stability. The compatibility of this instrumentation with established ‘pop-in’ diagnostic screens at (but not limited to) the entrance and exit of the SPB/SFX instrument provides an opportunity to differentiate between perturbations introduced by the accelerator and instrument optics. A sufficiently detailed parametric description of the source will have implications on the design of future experiments at the SPB/SFX instrument.

Efforts in the development of virtual diagnostics for the SPB/SFX instrument (Yoon *et al.*, 2016 ▸) should in future address observations obtained by MHz diagnostics. Contemporary parametric statistical models of XFEL radiation consider only fundamental properties originating from shot-noise: the spatial and temporal properties of XFEL photon pulses are assumed to be stochastic shot-to-shot (Pfeifer *et al.*, 2010 ▸). The systematic beam displacement and variation in beam size on inter-pulse timescales indicate that future numerical models of the source should consider pulse-perturbations that are time-evolving on the inter-pulse timescale. The shot-to-shot properties of the data presented in Figs. 4 ▸ and 5 ▸ suggest that, in future, the properties of pulses that share a pulse-train should not be assumed to be (i) statistically independent, or (ii) statistically stationary in space and time. This has significant implications in deriving a suitable correlation function to describe the similarity between inter-pulse intensity distributions (Manea, 2009 ▸), and likewise coherence measurements (Sereda *et al.*, 1998 ▸). We note that the field of cyclostationary random processes may be relevant to the study of statistically periodic pulse-train ensembles (Davis, 2007 ▸).

The recorded photon pulse properties are observed to be sensitive to beam trajectory and distribution. The inter-pulse drift varies significantly with the number of delivered pulses, encouraging further assessment of the impact of beam distribution on photon-beam stability. The measured sensitivity of the shot-to-shot beam properties to beam-trajectory modifications occurring in the accelerator indicates that existing electron-beam trajectory feedback systems can be used to remedy or constrain systematic structures in the inter-pulse properties of the beam. While a linear trajectory perturbation was applied to the horizontal component on the electron-beam trajectory, the perturbed pulse-trains exhibit greater fluctuations in beam-displacement in both transverse directions. Further efforts to calibrate the photon-beam response to applied trajectory modifications are necessary. The SASE1 undulator at the European XFEL is equipped with an upstream fast intra-bunch feedback system (IBFB) to reduce deviations from the nominal electron beam trajectory in the accelerator (Keil *et al.*, 2015 ▸). Future work will integrate MHz imaging diagnostics with upstream electron BPMs to create a diagnostic feedback loop that adjusts the electron-beam trajectory in the accelerator to improve the inter-pulse quality of the beam at the instrument.

## 6. Conclusion

We have demonstrated the application of novel MHz frame-rate photon beam diagnostics to the study of shot-to-shot pulse characteristics at the European XFEL. Our analysis has compared train- and pulse-integrated measurements across varying pulse distribution conditions as a route to improved parametric descriptions of the MHz source properties. We observed that train-integrated diagnostics are a poor reflection of the variability in pulse size and centre-of-mass as measured shot-to-shot. As an extension, we have shown that these diagnostics are sensitive to fast intra-bunch electron-beam trajectory feedback systems, which provide an opportunity for improving the quality of beam delivered to the instrument. We have identified unexpected structure in the shot-to-shot properties of the instrument-end beam that is not related to fundamental shot noise fluctuations and is reproducible between pulse-trains. Future applications of intra-train, two-dimensional diagnostics will address the implications of pulse-variability in an experimental context, seeking an improved understanding of the influence of source, accelerator and photon optics on perturbations in the two-dimensional intensity profile of the beam.

## Supplementary Material

Movie 1: Animation of the average inter-pulse beam profile for each pulse-position within a train in the 100 pulse-per-train beam distribution case. DOI: 10.1107/S1600577522005720/yi5126sup1.gif

Movie 2: Animation of the average inter-pulse beam profile for each pulse-position within a train in the 50 pulse-per-train beam distribution case. DOI: 10.1107/S1600577522005720/yi5126sup2.gif

Movie 3: Animation of the average train-integrated beam profile for each of the recorded pulse-trains in the 100 pulse-per-train beam distribution case. DOI: 10.1107/S1600577522005720/yi5126sup3.gif

## Figures and Tables

**Table 1:** Range and standard deviation of the horizontal and vertical beam displacement, and % change in beam size relative to the train-integrated, global and intra-train means; data were recorded for 76 and 170 trains in the 50 pulse-per-train and 100 pulse-per-train cases, respectively

Repetition rate	No. of pulses	Δ*x* (mm)	Δ*y* (mm)	Δ*r* (%)
10 Hz	50	0.356 ± 0.095	0.388 ± 0.078	18.4 ± 3.09
10 Hz	100	2.75 ± 0.302	0.280 ± 0.054	30.6 ± 3.88
564 kHz (all pulses)	50	1.13 ± 0.144	0.525 ± 0.077	43.5 ± 5.19
1.128 MHz (all pulses)	100	3.29 ± 0.338	0.727 ± 0.102	42.8 ± 6.83
564 kHz (intra-train)	50	0.539 ± 0.098	0.190 ± 0.029	17.4 ± 2.78
1.128 MHz (intra-train)	100	0.699 ± 0.083	0.424 ± 0.061	25.5 ± 3.60

**Table 2:** Global and intra-train average range and standard deviation in beam properties corresponding to the perturbed and unperturbed electron bunch trains

	Perturbation	Δ*x* (mm)	Δ*y* (mm)	Δ*r* (%)
1.128 MHz (all pulses)	Y	0.83 ± 0.23	0.52 ± 0.13	23.63 ± 6.27
	N	0.48 ± 0.09	0.31 ± 0.05	21.30 ± 3.43
1.128 MHz (intra-train)	Y	0.71 ± 0.05	0.42 ± 0.04	19.72 ± 1.30
	N	0.31 ± 0.04	0.20 ± 0.02	13.13 ± 1.48
