# Supplementary figures and images for: Shot-to-shot two-dimensional photon intensity diagnostics within megahertz pulse-trains at the European XFEL
Source: J Synchrotron Radiat. 2022 Jun 8;29(Pt 4):939–46. doi: 10.1107/S1600577522005720 (PMC9255581; doi:10.1107/S1600577522005720)

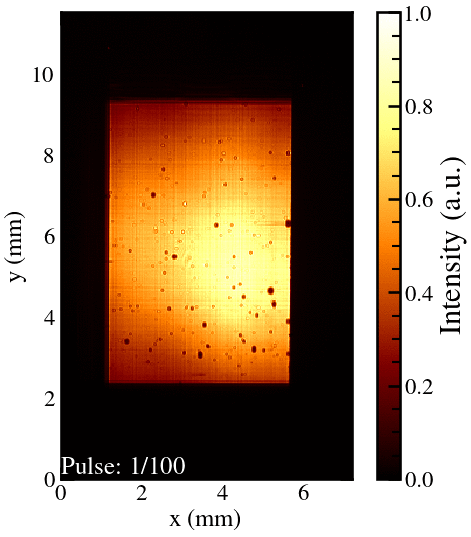

Supplement: Supplementary file 1 [file s-29-00939-sup1.gif]

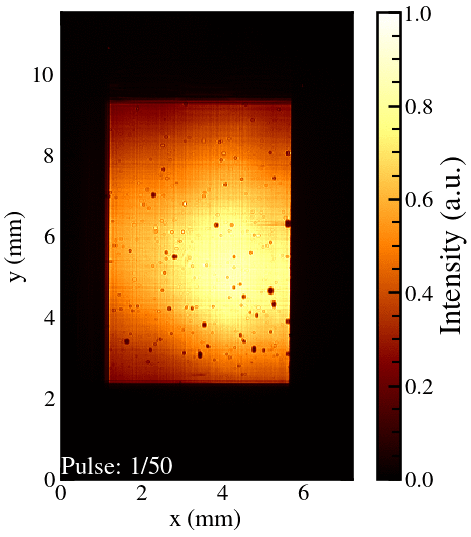

Supplement: Supplementary file 2 [file s-29-00939-sup2.gif]

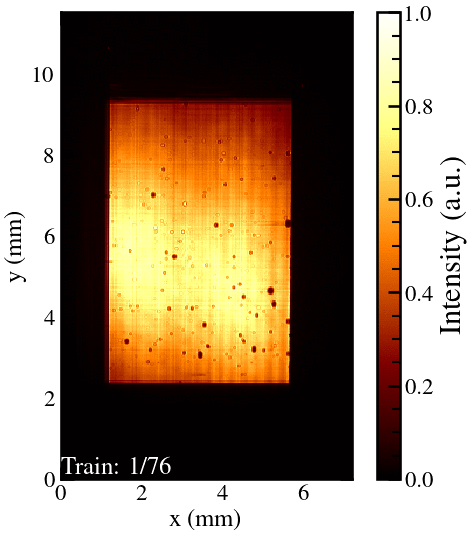

Supplement: Supplementary file 3 [file s-29-00939-sup3.gif]
